# A Markov Chain Monte Carlo (MCMC) Multivariate Analysis of the Association of Vital Parameter Variation With the Lunar Cycle in Patients Hospitalized With COVID-19

**DOI:** 10.7759/cureus.34290

**Published:** 2023-01-27

**Authors:** Supriya Koya, Sreeja Ponnam, Sharon Salenius, Sayanu Pamidighantam

**Affiliations:** 1 Hematology and Oncology, GenesisCare, Ponca City, USA; 2 Hematology and Medical Oncology, Hillcrest Medical Center, Tulsa, USA; 3 Internal Medicine, University of Missouri - Kansas City School of Medicine, Kansas City, USA; 4 Research and Education, GenesisCare, Fort Myers, USA; 5 Physics, Birla Institute of Technology and Science, Hyderabad, IND

**Keywords:** vital parameter destabilization window, circadian rhythms, lunar phase, non-covid, covid, vitals, lunar cycle

## Abstract

Introduction: Over the last three years, the world has been battling a long-drawn pandemic resulting from the coronavirus outbreak. Despite the safety measures, there have been multiple pandemic waves happening throughout the world. Therefore, it is necessary to understand the fundamental characteristics of COVID-19 transmission and pathogenesis to overcome the threat of the pandemic. This study focused on hospitalized COVID-19 patients because of their high mortality rate, which indicates the need to improve inpatient management.

Methods: Based on the cyclic nature of the pandemic, observations were made to examine the influence of lunar phases on six vital parameters of COVID-19 patients. A multivariate analysis was carried out to study the interactions of lunar phase pairwise on COVID-19 statuses and COVID-19 status pairwise on lunar phases by treating six vital parameters as independent entities.

Results: The results of multivariate analysis on the data of 215,220 vital values showed that lunar phases are associated with trends in variations in the vital parameters of COVID-19-infected patients.

Conclusion: In summary, our results show that patients infected with COVID-19 appear to be more susceptible to lunar influence compared to non-COVID-19 patients. Furthermore, this study shows a vital parameter destabilization window (DSW) that can help identify which hospitalized COVID-19 patients can recover. Our pilot study forms the basis for future studies to eventually establish the incorporation of variation of vital signs with the lunar cycle into the standard of care for COVID-19 patients.

## Introduction

Pandemics from coronaviruses have occurred multiple times in the past few decades. Since its emergence in the form of severe acute respiratory syndrome (SARS) in 2002, Middle East respiratory syndrome (MERS) in 2012, and severe acute respiratory syndrome coronavirus 2 (SARS-CoV-2) in 2019, variations have been occurring more frequently over time with the current virus stronger than ever before [1]. Worldwide, over 663 million individuals have been infected with COVID-19, and more than 6.6 million deaths have been registered to date [2]. The United States alone accounts for 100 million of those infections and more than one million deaths from COVID-19 at the time of writing this article [2]. Multiple studies reporting on mask mandates indicate that wearing masks can reduce COVID-19-related infections and deaths to an extent [3]. While masks, social distancing, and vaccinations help in controlling the spread of the virus, these measures alone cannot prevent the surges of the virus completely. This is evidenced by the emergence of new mutants such as the Omicron variants and the vulnerability of vaccinated people against the new variants. Despite the safety measures taken, there have been multiple pandemic waves occurring worldwide. Therefore, it is necessary to understand the fundamental characteristics of COVID-19 transmission to overcome the threat of the pandemic.

Lunar influence on the occurrence of cardiovascular and cardiopulmonary events has been reported in the literature [4-6]. Prior research has been performed on lunar influence on the vital signs of healthy subjects [7], and there are also publications on calculating prognostic nomograms based on vital parameters in COVID-19 patients [8-10]. To the best of our knowledge, we believe this is the first study reporting lunar influence on the vital signs of hospitalized COVID-19 and non-COVID-19 patients using multivariate analysis. The purpose of this study is to examine our hypotheses listed as follows: lunar phases have a significant effect on the vital signs of hospitalized COVID-19 patients, patients hospitalized with COVID-19 have higher rates of vital sign fluctuations during lunar phases than patients hospitalized without COVID-19, and a “vital parameter destabilization window (DSW)” can predict the severity of COVID-19 infection in hospitalized patients.

A pilot study to validate the proposed hypotheses is performed. Multivariate statistical analysis was performed on the vital signs of COVID-19-infected patients (both alive and deceased) in relation to the lunar phases. As a control group, patients hospitalized for non-COVID-19-related diagnoses were included. Finally, based on the cyclic nature of the pandemic, observations were made to examine the influence of lunar phases on the six vital parameters of COVID-19 patients.

## Materials and methods

**Figure 1:**
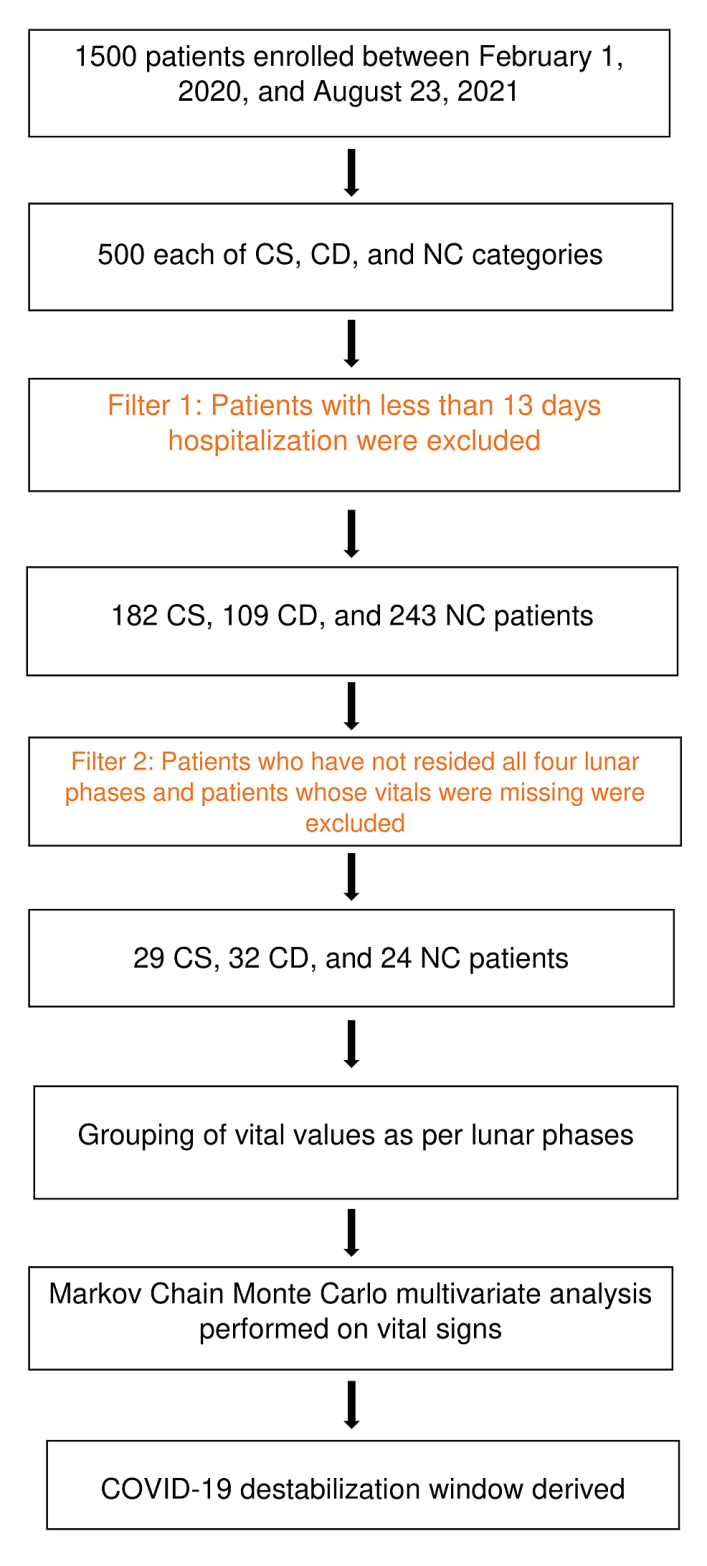
Schematic representation of the study design COVID-19: coronavirus disease 2019, CS: patients hospitalized with COVID-19 who survived, CD: patients hospitalized with COVID-19 who are deceased from COVID-19, NC: patients hospitalized for non-COVID-19 diagnosis

Upon approval from the Hillcrest Medical Center Institutional Review Board (IRB), patients admitted between February 1, 2020, and August 23, 2021, at Hillcrest Health System in Northeast Oklahoma with a diagnosis code of COVID-19 were identified. A total of 1000 patient samples were gathered for the pilot study. Among these were 500 patients hospitalized with COVID-19 who survived COVID-19 infection (CS) and 500 patients hospitalized with COVID-19 who died from COVID-19 infection (CD). Applying hospitalization criteria of at least 13 days, the sample size was reduced to 182 CS and 109 CD patients; 13 days were chosen so that the cohorts will have enough vitals in each of the lunar phases. All these patients were confirmed positive for COVID-19 based on COVID-19 PCR tests and symptoms. In addition, we have taken 243 patients hospitalized for other reasons between February 1, 2020, and August 23, 2021, who did not have COVID-19 (non-COVID-19 patients (NC)) as benchmark or control subjects. Patients who did not have vitals during most of the four moon phases were excluded, resulting in a sample size of 29 CS, 32 CD, and 24 NC patients. All these 85 patients have vital signs data corresponding to the four lunar phases. These 85 patients had 215,220 observations or vital sign data. This study design schema is depicted in Figure 1.

A lunar month is defined as the duration between the day after the new moon to the beginning of the next new moon. The vital signs data of these patients were divided into four moon phases: new moon (NM), half-moon after new moon or waxing moon or first quarter (FQ), full moon (FM), and half-moon after the full moon or waning moon or third quarter (TQ). Data on lunar phases and times are taken from the lunar calendar [11]. The Oklahoma time zone was used while grouping the vital parameter data among the four lunar phases.

For the purpose of the current article, we present a multivariate analysis of vital signs in relation to lunar phases for 29 CS, 32 CD, and 24 NC patients. Since these were all hospitalized patients, vital signs were continuously monitored via automatic systems. Systolic blood pressure (SYSBP) and diastolic blood pressure (DIABP), heart rate (PULSE), respiratory rate (RESP), pulse oximetry (PULSEOX), and temperature (TEMP) were measured approximately a minimum of every 15 minutes to a maximum of every eight hours depending on the severity of the patient’s condition, while the temperature was monitored approximately a minimum of every four hours to a maximum of 12 hours. Examination of raw data revealed a number of anomalous values outside the range of reasonably possible values in the vital sign data that may indicate that the patient was deceased or some measurement error occurred. It was decided to restrict the range of values analyzed to the following: 30<𝐷𝐼𝐴𝐵𝑃<200, 25<𝑃𝑈𝐿𝑆𝐸<200, 50<𝑃𝑈𝐿𝑆𝐸𝑂𝑋≤100, 6<𝑅𝐸𝑆𝑃<50, 50< 𝑆𝑌𝑆𝐵𝑃<300, and 82<𝑇𝐸𝑀𝑃<107.

We used a multivariate mixed model (multivariate analysis) [12,13]. The main reason for choosing a mixed model for this analysis is that many repeated observations of each vital sign were taken for each patient. Observations close together in time will not be independent, which is confirmed by the box plots in the supplemental figure (Appendices), which show that median first-order autocorrelations are quite high (>0.6) for all vital sign measures. A mixed model allows this within-subject correlation to be accounted for, thus avoiding bias in results due to dependence among the observations. A second source of dependence arises from the fact that six vital sign measures were taken for each patient. These repeated measures will also be correlated within each patient as is shown in the correlation matrix shown in the supplemental table (Appendices). A Markov chain Monte Carlo (MCMC) multivariate mixed model [12,13] with random intercepts to account for both correlation between variables and the within-patient correlation over time was used to model the response for each of the six vital variables. The model is as follows: 𝑌=𝑉+𝐿∗𝑉+𝐶∗𝑉+𝐿∗𝐶∗𝑉+𝑈𝐼𝐷+𝐸, where Y contains the responses for the six vital sign variables, V is a factor with six levels indexing the six variables, L is a factor with four levels indexing the four main phases of the moon, C is a factor with three levels indexing COVID-19 status, UID is a random effect allowing for different intercepts for each patient, and E is the random error. This model allows for the expected differences in means between the six variables and the estimation of lunar phase and COVID-19 status effects along with their interaction effects for each of the six variables. The random intercepts and error terms allow for the estimation of the within-patient correlation. This model does not include the demographic and comorbidity factors.

## Results

Between February 1, 2020, and August 23, 2021, the median number of observations (vital signs) per patient is 2,406, with 95% of patients having between 266 and 5,344 observations. The median collection interval was 15 minutes, with 95% of intervals falling between five and 60 minutes. The total number of records of vital signs in the raw data set is 215,220.

Table 1 shows the demographic and clinical characteristics of the patients. Older age individuals, male gender, patients on ventilators, and extracorporeal membrane oxygenation (ECMO) are showing more variability in their vital signs (more fluctuations from normal range) with respect to lunar phases (p<0.005). This is in line with descriptive studies showing that men are more susceptible to COVID-19 [14] and older age individuals have a high case fatality rate [15].

**Table 1:** Demographic and clinical characteristics of the 85 patients COVID-19: coronavirus disease 2019

Characteristics	Values	Number	Percent
Gender	Female	35	41.2%
	Male	50	58.8%
Race	White	48	56.5%
	Black	13	15.3%
	Hispanic	4	4.7%
	Native American	17	20%
	Other	3	3.5%
Any comorbidity	No	3	3.5%
	Yes	82	96.5%
Diabetes mellitus	No	59	69.4%
	Yes	26	30.6%
Any cardiac condition	No	44	51.8%
	Yes	41	48.2%
Any psychiatric condition	No	69	81.2%
	Yes	16	18.8%
Had COVID-19 vaccine	No	73	86.9%
	Yes	11	13.1%
Placed on ventilator	No	36	42.4%
	Yes	49	57.6%
Placed on extracorporeal membrane oxygenation	No	58	68.2%
	Yes	27	31.8%
Patient obese	No	42	49.4%
	Yes	43	50.6%

Analyzing the data from Table 1 using the model presented showed that DIABP variability with lunar phases is statistically significant with respect to age (p=0.021) and being placed on a ventilator (p=0.018), PULSEOX variability with lunar phases is statistically significant with respect to gender (p=0.014) and age (p=0.013), RESP variability with lunar phases is statistically significant with respect to being placed on a ventilator (p=0.001) and ECMO (p=0.002), PULSE variability with lunar phases is significant with respect to age (p=0.001), and SYSBP variability with lunar phases is significant with respect to being placed on ECMO (p=0.041).

**Figure 2:**
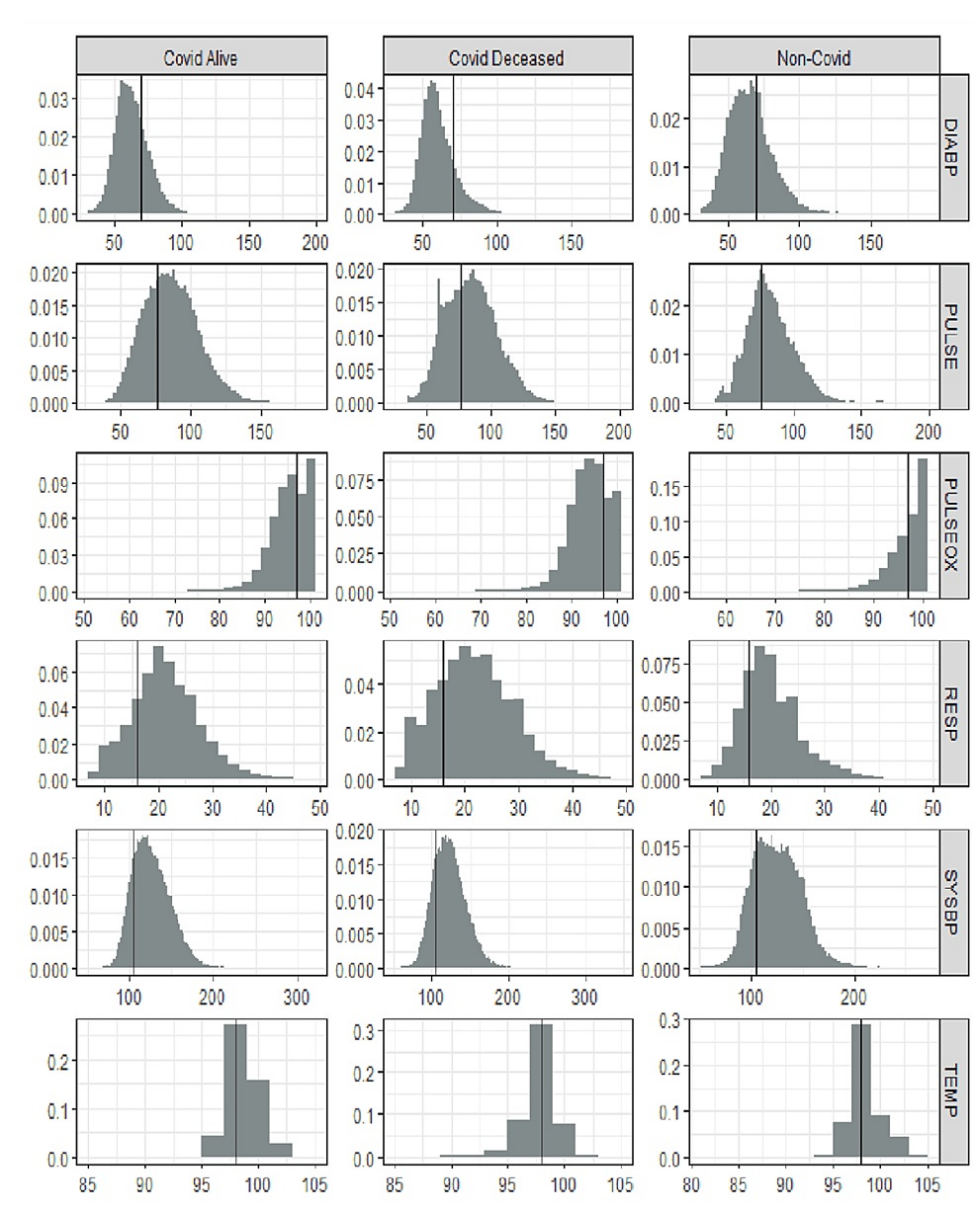
Histograms of the distribution of six vitals across all patients in the study Histograms indicating 2%-7% of total vital sign observations are in the normal range. COVID: coronavirus disease, DIABP: diastolic blood pressure, PULSE: heart rate, PULSEOX: pulse oximetry, RESP: respiratory rate, SYSBP: systolic blood pressure, TEMP: temperature

Most vital parameters are out of the medically accepted normal range in all three categories for most patients (Figure 2). All variables, except PULSEOX, exhibit relatively normal (bell-shaped) distributions with a slight right skew in the distributions of DIABP, PULSE, RESP, and SYSBP. Of the total vital signs’ observations, 2%-7% are in the normal range in this cohort of hospitalized patients, and 93%-98% of the vital signs’ observations are outside the normal window range (Figure 2).

Figure 2 also shows PULSEOX being in the normal range at 10% of the total patient observations compared to other parameters such as DIABP, which is in the normal range at 2.5% of the total observations. PULSEOX variability with the lunar phase is statistically significant with respect to gender and age (Table 1), which means that the lunar cycle has a larger impact on PULSEOX compared to other vitals, and it is varying more than other vital parameters with each lunar phase.

The medically defined normal mean values for vital signs are as follows: DIABP, 70 mmHg; PULSE, 76 minute-1; PULSEOX, 97%; RESP, 16 minute-1; SYSBP, 105 mmHg; and TEMP, 98°F. We observed that DIABP mean values are less than normal; PULSE, RESP, and SYSBP mean values are above normal; and TEMP and PULSEOX mean values have mixed behavior with respect to the moon phases in all three sets of patients. For DIABP, SYSBP, TEMP, and PULSEOX, the mean values of CD across four lunar phases are lower than those of CS and NC. For RESP, the mean values of CD are higher than those of CS and NC. For PULSE, the mean values of CS are higher than those of CD and NC. This is represented in Table 2.

**Table 2:** Mean and standard errors for the vital signs at each phase of the moon and COVID-19 statuses COVID-19: coronavirus disease 2019, new: new moon, waxing: waxing moon or first quarter (FQ), full: full moon, waning: waning moon or third quarter (TQ), DIABP: diastolic blood pressure, PULSE: heart rate, PULSEOX: pulse oximetry, RESP: respiratory rate, SYSBP: systolic blood pressure, TEMP: temperature, SE: standard error

		New		Waxing		Full		Waning	
		Mean	SE	Mean	SE	Mean	SE	Mean	SE
DIABP	COVID-19 alive	63.75	0.078	64.51	0.078	64.92	0.084	63.43	0.086
	COVID-19 deceased	60.79	0.076	60.45	0.082	61.47	0.089	60.78	0.076
	Non-COVID-19	67.34	0.160	65.97	0.173	66.62	0.160	66.29	0.159
PULSE	COVID-19 alive	85.31	0.128	86.15	0.124	88.37	0.129	87.14	0.115
	COVID-19 deceased	84.09	0.129	84.25	0.149	84.13	0.125	87.47	0.143
	Non-COVID-19	81.11	0.176	81.35	0.179	85.30	0.195	85.72	0.164
PULSEOX	COVID-19 alive	96.16	0.025	95.51	0.025	95.26	0.026	95.75	0.027
	COVID-19 deceased	94.44	0.029	94.32	0.031	94.19	0.030	94.31	0.030
	Non-COVID-19	97.20	0.033	96.88	0.049	97.40	0.036	97.33	0.036
RESP	COVID-19 alive	21.87	0.044	21.70	0.042	22.24	0.038	21.70	0.040
	COVID-19 deceased	21.99	0.049	22.01	0.052	23.29	0.048	22.22	0.049
	Non-COVID-19	21.98	0.070	19.63	0.056	19.99	0.049	20.45	0.053
SYSBP	COVID-19 alive	126.76	0.141	124.08	0.136	129.18	0.162	128.36	0.152
	COVID-19 deceased	124.68	0.151	124.28	0.150	124.83	0.155	121.12	0.143
	Non-COVID-19	127.25	0.260	126.66	0.255	126.18	0.251	124.70	0.220
TEMP	COVID-19 alive	98.92	0.009	98.70	0.009	98.74	0.008	98.55	0.007
	COVID-19 deceased	97.57	0.012	97.99	0.007	98.28	0.008	97.78	0.008
	Non-COVID-19	98.50	0.016	97.93	0.012	98.17	0.015	99.02	0.019

Taking into account medically defined normal mean values for vital signs as DIABP of 70 mmHg, PULSE of 76 minute-1, 97% PULSEOX, RESP of 16 minute-1, SYSBP of 105 mmHg, and TEMP of 98°F and subtracting this normal value from the cumulative mean of all four lunar phase vital parameters and dividing by the normal value, we were able to get the extent of deviation from the normal as shown in Table 3.

**Table 3:** The derived extent of deviation from the normal value of the mean of all vitals across all lunar phases for three sets of COVID-19 patients COVID-19: coronavirus disease 2019, DIABP: diastolic blood pressure, PULSE: heart rate, PULSEOX: pulse oximetry, RESP: respiratory rate, SYSBP: systolic blood pressure, TEMP: temperature

Vital	COVID-19 status	New moon mean	Waxing moon mean	Full moon mean	Waning moon mean	Total mean value	Percent deviation from normal
DIABP	COVID-19 alive	63.75	64.51	64.92	63.43	64.15	-8.30%
	COVID-19 deceased	60.79	60.45	61.47	60.78	60.87	-13.03%
	Non-COVID-19	67.34	65.97	66.62	66.29	60.55	-4.92%
PULSE	COVID-19 alive	85.31	86.15	88.37	87.14	86.74	14.13%
	COVID-19 deceased	84.09	84.25	84.13	87.47	84.98	11.82%
	Non-COVID-19	81.11	81.35	85.3	85.72	83.37	9.69%
PULSEOX	COVID-19 alive	96.16	95.51	95.26	95.75	95.67	-1.37%
	COVID-19 deceased	94.44	94.32	94.19	94.31	94.31	-2.70%
	Non-COVID-19	97.2	96.88	97.4	97.33	97.2	-0.20%
RESP	COVID-19 alive	21.87	21.7	22.24	21.7	21.87	36.73%
	COVID-19 deceased	21.99	22.01	23.29	22.22	22.37	39.85%
	Non-COVID-19	21.98	19.63	19.99	20.45	20.51	28.20%
SYSBP	COVID-19 alive	126.76	124.08	129.18	128.36	127.09	21.04%
	COVID-19 deceased	124.68	124.28	124.83	121.12	123.72	17.83%
	Non-COVID-19	127.25	126.66	126.18	124.7	126.19	20.18%
TEMP	COVID-19 alive	98.92	98.7	98.74	98.55	98.72	-0.74%
	COVID-19 deceased	97.57	97.99	98.28	97.78	97.9	-0.09%
	Non-COVID-19	98.5	97.93	98.17	99.02	98.4	-0.41%

For CD, the cumulative mean of DIABP across four lunar phases is below normal (-13%), PULSEOX cumulative mean value is below normal (-2.70%), TEMP cumulative mean value is below normal (-0.09%), SYSBP cumulative mean value is above normal (+17.83%), PULSE cumulative mean value is above normal (+11.82%), and RESP cumulative mean value is above normal (+39.85%). For CS, the cumulative mean value of DIABP is below normal (-8.30%), PULSEOX cumulative mean value is below normal (-1.37%), TEMP cumulative mean value is below normal (-0.74%), SYSBP cumulative mean value is above normal (+21.04%), PULSE cumulative mean value is above normal (+14.13%), and RESP cumulative mean value is above normal (+36.73%). For NC, the cumulative mean value of DIABP is below normal (-4.92%), PULSEOX cumulative mean value is below normal (-0.21%), TEMP cumulative mean value is below normal (-0.41%), SYSBP cumulative mean value is above normal (+20.18%), PULSE cumulative mean value is above normal (+9.69%), and RESP cumulative mean value is above normal (+28.2%).

**Figure 3:**
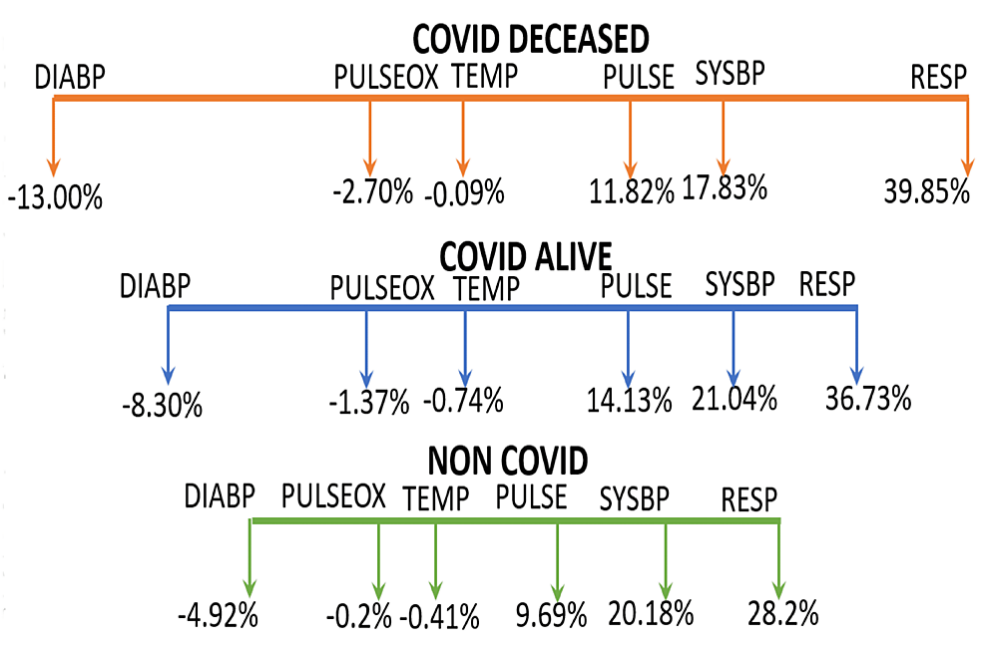
Vital parameter DSW for three categories for CD, CS, and NC DSW: destabilization window, CD: COVID-19 deceased, CS: COVID-19 survived, NC: non-COVID-19, DIABP: diastolic blood pressure, PULSEOX: pulse oximetry, TEMP: temperature, PULSE: heart rate, SYSBP: systolic blood pressure, RESP: respiratory rate

Based on these data, a window termed a vital parameter destabilization window (DSW) was deduced. We observed that the maximum and minimum vital parameter variation with respect to the normal value as a function of moon phases is between -13% and +39.9% in CD, -8.3% and +36.7% in CS, and -4.9% and +28.2% in NC. Figure 3 shows the vital parameter DSW for CD, CS, and NC patients as a function of moon phases.

**Figure 4:**
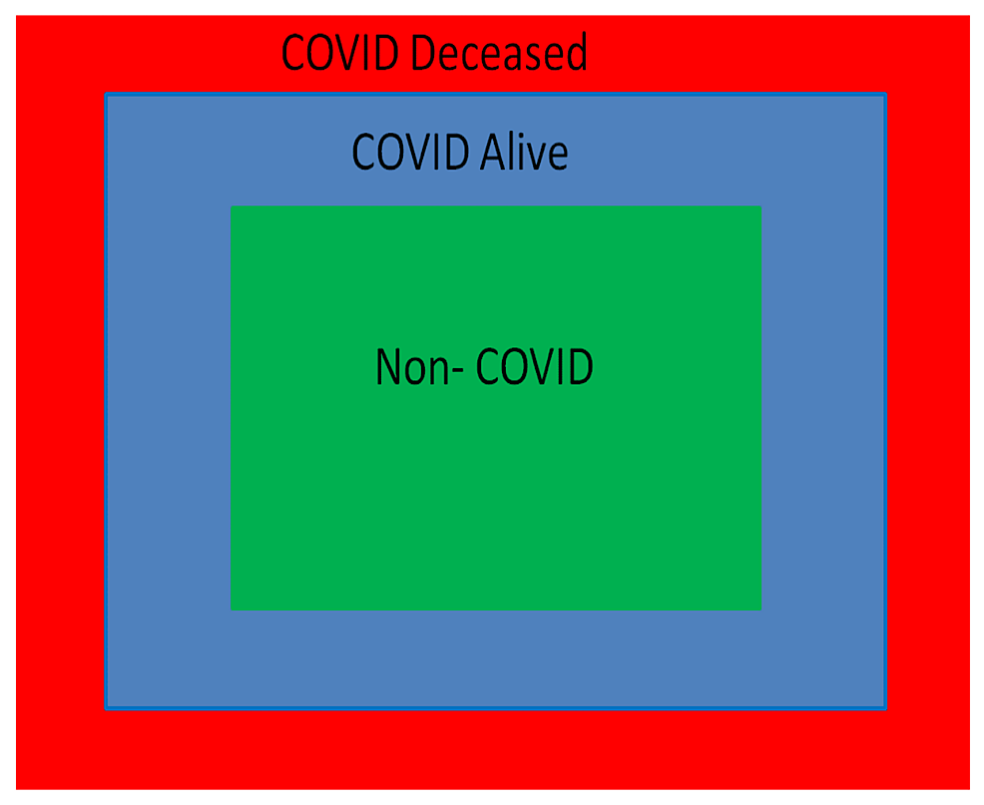
Schematic of vital parameter DSW At the overlap of green and blue zones, recovery from COVID-19 is observed. DSW: destabilization window, COVID-19: coronavirus disease 2019

Schematically representing DSW as red color for CD, blue color for CS, and green color for NC, it can be seen that at the overlap of green and blue color, recovery from COVID-19 is observed. This is represented in Figure 4.

Data in Table 3 were taken and plotted in graphs for three categories (CD, CS, and NC) of all vitals with respect to moon phases by taking the percent deviation of the mean from medically defined normal values.

**Figure 5:**
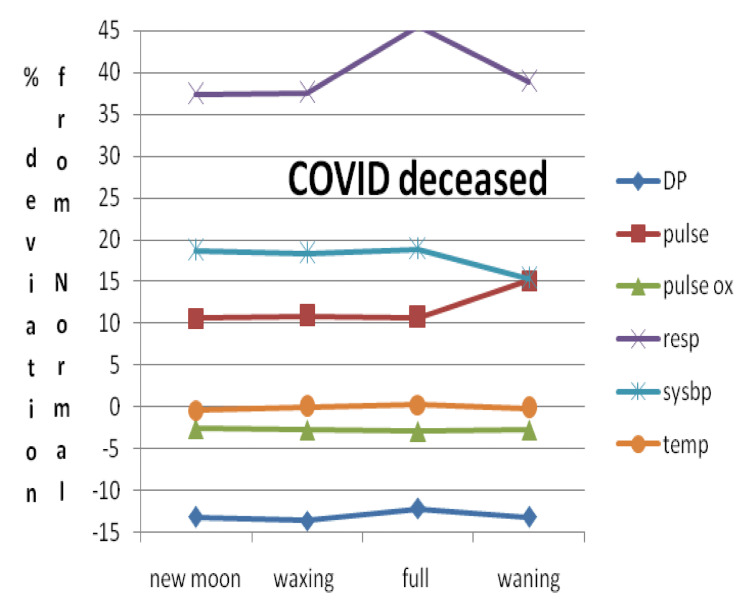
Vital parameter DSW with respect to moon phases for CD plotted by taking the percent deviation of the mean from the medically defined normal values DSW: destabilization window, CD: COVID-19 deceased, COVID: coronavirus disease, DP: diastolic blood pressure, pulse: heart rate, pulse ox: pulse oximetry, resp: respiratory rate, sysbp: systolic blood pressure, temp: temperature

DSW with respect to moon phases for CD plotted by taking the percent deviation of the mean from medically defined normal values is shown in Figure 5.

**Figure 6:**
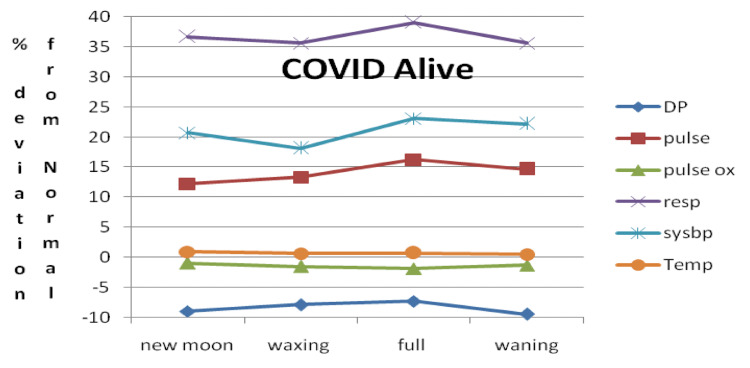
Vital parameter DSW with respect to moon phases for CS plotted by taking the percent deviation of the mean from medically defined normal values DSW: destabilization window, CS: COVID-19 survived, COVID: coronavirus disease, DP: diastolic blood pressure, pulse: heart rate, pulse ox: pulse oximetry, resp: respiratory rate, sysbp: systolic blood pressure, temp: temperature

DSW with respect to moon phases for CS plotted by taking the percent deviation of the mean from medically defined normal values is shown in Figure 6.

**Figure 7:**
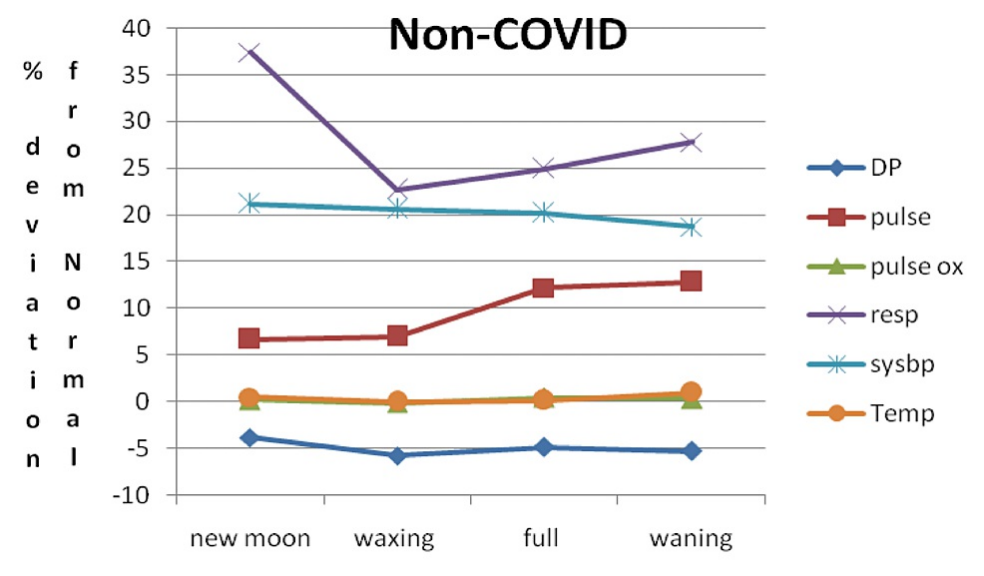
Vital parameter DSW with respect to moon phases for NC plotted by taking the percent deviation of the mean from medically defined normal values DSW: destabilization window, NC: non-COVID-19, COVID: coronavirus disease, DP: diastolic blood pressure, pulse: heart rate, pulse ox: pulse oximetry, resp: respiratory rate, sysbp: systolic blood pressure, temp: temperature

DSW with respect to moon phases for NC plotted by taking the percent deviation of the mean from medically defined normal values is shown in Figure 7.

Our observation has shown that when the values for DIABP and RESP deviate from the normal window by -13.1% and 39.9% over one lunar month cycle, it can be inferred that the patient’s health condition is critical and they are unable to compensate by returning to their homeostasis, thus eventually succumbing to COVID-19. In contrast, when the DIABP and RESP values deviate from the normal operation window by -8.36% and 36.7%, respectively, COVID-19 recovery is possible. Finally, when the DIABP and RESP values deviate from normal by -4.92% and 28.2% over a lunar month cycle, not only is recovery possible but also the patient likely has better immunity toward COVID-19 and thus is not infected with COVID-19 (Figure 3-7). If we consider the non-COVID-19 destabilization window as unity, then the COVID-19 alive destabilization window is as large as 1.36, and the COVID-19 deceased destabilization window is further larger by 1.6.

The multivariate F tests that were conducted are statistically significant as shown in Table 4. The variable: lunarphase4 and variable:covidstatus effects are both highly significant (p<0.001), indicating that significant differences between lunar phases and COVID-19 statuses exist for at least one of the vital sign variables. The variable:lunarphase4:covidstatus interaction effect is significant (p<0.001), indicating that for at least one vital sign measure, the effect of the lunar phase differs across levels of COVID-19 status. The nature of this effect is explored further by examining lunar phase pairwise differences for each COVID-19 status and vital sign.

**Table 4:** Multivariate F-tests and p-values for the fixed effects in model 1 Note: df2 = Inf simply means that the numerator “degrees of freedom” was so large that a normal approximation was used to calculate p-values. V: variable, L: lunar phase, C: COVID-19 status, df: degrees of freedom

Terms in model	df1	df2	F ratio	p-value
V	5	Inf	12990.3	0.000
variable:lunarphase4 (L*V)	18	Inf	71.0	0.000
variable:covidstatus (C*V)	12	Inf	7.4	0.000
variable:lunarphase4:covidstatus (L*C*V)	36	Inf	113.621	0.000

**Figure 8:**
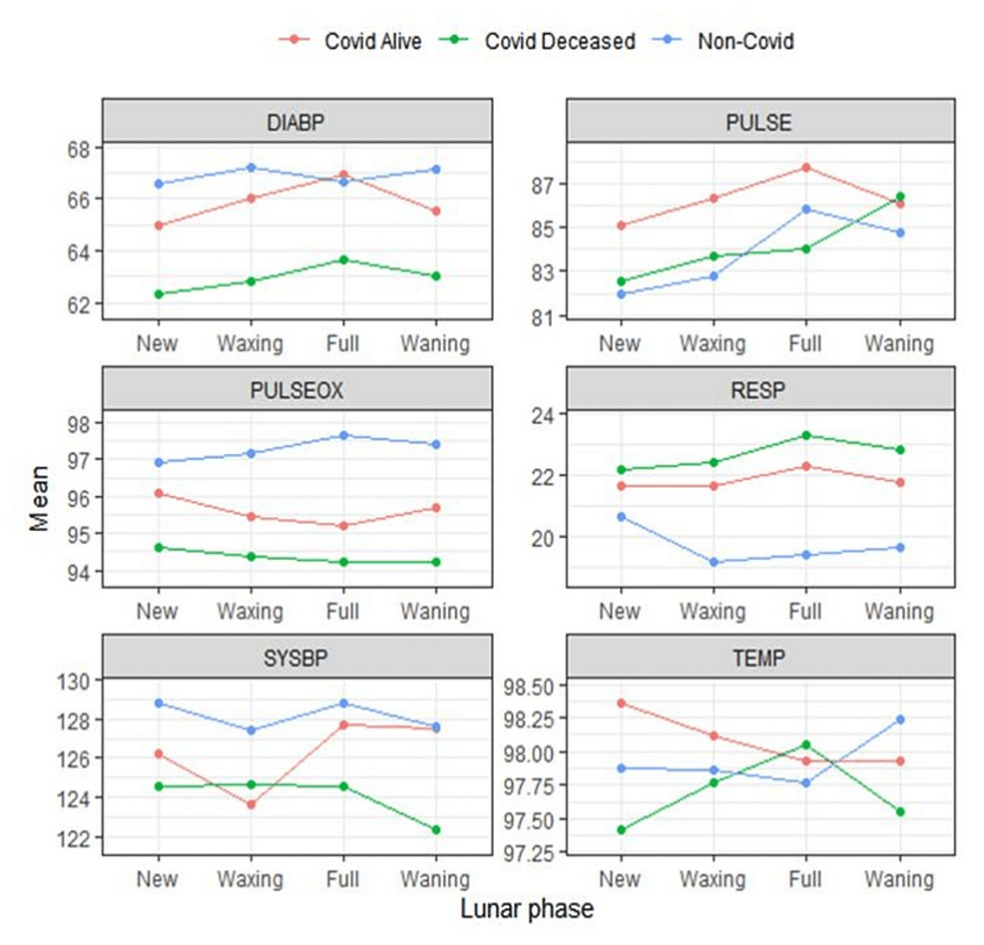
Lunar phase and COVID-19 status interaction plots by vital signs COVID-19: coronavirus disease 2019, DIABP: diastolic blood pressure, PULSE: heart rate, PULSEOX: pulse oximetry, RESP: respiratory rate, SYSBP: systolic blood pressure, TEMP: temperature

Since the lunar phase and COVID-19 status interaction is significant, their effects need to be examined jointly as shown in Figure 8. Figure 8 shows the interaction plots of these factors for each vital sign where the extent of interaction is indicated by the concordance of line profiles for each COVID-19 status. If the profiles are parallel, the effect of the lunar phase is the same for each COVID-19 status. If the profiles are not parallel, the effect of the lunar phase differs for at least one COVID-19 status. For example, the profiles for temperature are not parallel, indicating that any effect of COVID-19 status depends on which lunar phase we are considering. In this case, we could say that the mean temperature at the new moon is lower for COVID-19 deceased patients than for non-COVID-19 and COVID-19 alive patients, whereas at full moon, there is likely no difference in mean temperature between COVID-19 statuses.

Lunar phase by COVID-19 status pairwise comparisons

**Figure 9:**
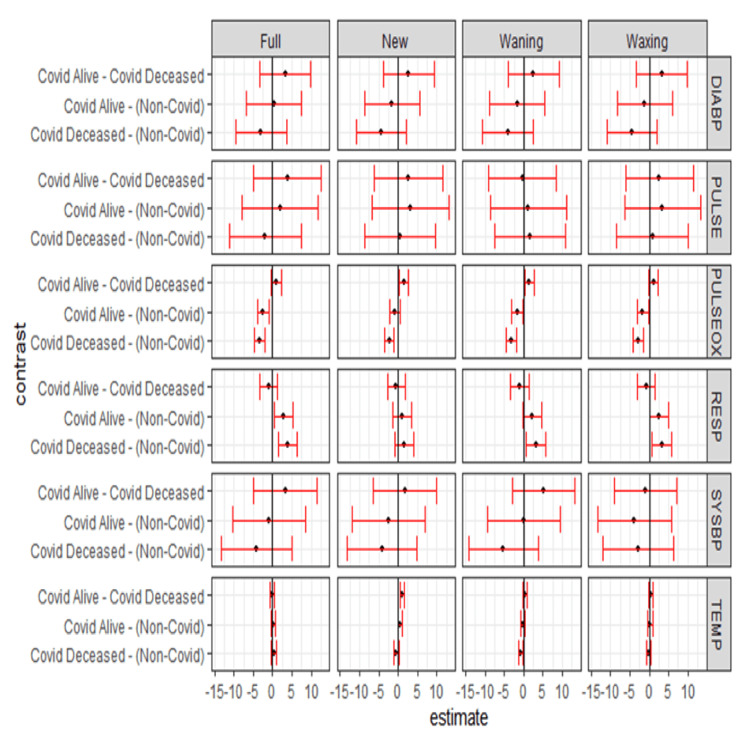
COVID-19 status pairwise effect plots by lunar phase and vital sign Confidence intervals are simultaneous at 95% as they have been adjusted using the multivariate T-distribution method. COVID: coronavirus disease, DIABP: diastolic blood pressure, PULSE: heart rate, PULSEOX: pulse oximetry, RESP: respiratory rate, SYSBP: systolic blood pressure, TEMP: temperature

The plots in Figure 9 display the pairwise contrast estimates and simultaneous confidence intervals for pairwise differences (contrasts) between lunar phases by COVID-19 status and vital signs [16-18]. If an interval does not contain 0 (vertical line in plots), the contrast is statistically significant. Multivariate mixed method analysis of different categories of COVID-19 patient cohorts by lunar phases and vital signs are depicted in Figure 9. As can be seen, CD and NC group PULSEOX and RESP vital sign variation with respect to the lunar phase was significant (not crossing one, staying either on the right side or the left side).

The associated numerical estimates and multiple comparison-adjusted p-values are given in Table 5. As seen in Table 5, 75.9% of the p-values of the lunar phase pairwise show a statistical significance of less than 0.05.

**Table 5:** Lunar phase pairwise comparisons by COVID-19 status for all vital signs p-values are adjusted for comparisons using the multivariate T-distribution method. COVID-19: coronavirus disease 2019, DIABP: diastolic blood pressure, PULSE: heart rate, PULSEOX: pulse oximetry, RESP: respiratory rate, SYSBP: systolic blood pressure, TEMP: temperature

		COVID-19 alive		COVID-19 deceased		Non-COVID-19	
Vital sign	Contrast	Est.	p	Est.	p	Est.	p
DIABP	New - waxing	-1.043	0.000	-0.492	0.005	-0.619	0.136
	New - full	-1.907	0.000	-1.359	0.000	-0.039	1.000
	New - waning	-0.500	0.001	-0.712	0.000	-0.520	0.359
	Waxing - full	-0.863	0.000	-0.867	0.000	0.580	0.335
	Waxing - waning	0.543	0.000	-0.220	0.994	0.099	1.000
	Full - waning	1.406	0.000	0.647	0.000	-0.481	0.677
PULSE	New - waxing	-1.206	0.000	-1.183	0.000	-0.841	0.064
	New - full	-2.662	0.000	-1.511	0.000	-3.877	0.000
	New - waning	-0.945	0.000	-3.933	0.000	-2.806	0.000
	Waxing - full	-1.455	0.000	-0.328	0.945	-3.036	0.000
	Waxing - waning	0.262	0.997	-2.75	0.000	-1.965	0.000
	Full - waning	1.717	0.000	-2.422	0.000	1.071	0.003
PULSEOX	New - waxing	0.616	0.000	0.233	0.000	-0.213	0.015
	New - full	0.864	0.000	0.386	0.000	-0.740	0.000
	New - waning	0.357	0.000	0.4	0.000	-0.485	0.000
	Waxing - full	0.247	0.000	0.154	0.011	-0.527	0.000
	Waxing - waning	-0.259	0.000	0.167	0.002	-0.271	0.000
	Full - waning	-0.507	0.000	0.014	1.000	0.255	0.002
RESP	New - waxing	0.019	1.000	-0.236	0.078	1.451	0.000
	New - full	-0.634	0.000	-1.114	0.000	1.229	0.000
	New - waning	-0.135	0.924	-0.638	0.000	0.987	0.000
	Waxing - full	-0.653	0.000	-0.878	0.000	-0.222	0.909
	Waxing - waning	-0.154	0.601	-0.403	0.000	-0.464	0.000
	Full - waning	0.499	0.000	0.475	0.000	-0.242	0.762
SYSBP	New - waxing	2.642	0.000	-0.097	1.000	1.302	0.047
	New - full	-1.493	0.000	0.012	1.000	-0.063	1.000
	New - waning	-1.239	0.000	2.198	0.000	1.150	0.091
	Waxing - full	-4.135	0.000	0.11	1.000	-1.365	0.024
	Waxing - waning	-3.881	0.000	2.296	0.000	-0.152	1.000
	Full - waning	0.254	1.000	2.186	0.000	1.213	0.049
TEMP	New - waxing	0.256	0.000	-0.356	0.000	0.014	1.000
	New - full	0.435	0.000	-0.632	0.000	0.105	0.233
	New - waning	0.437	0.000	-0.138	0.000	-0.363	0.000
	Waxing - full	0.179	0.000	-0.276	0.000	0.090	0.874
	Waxing - waning	0.182	0.000	0.218	0.000	-0.377	0.000
	Full - waning	0.003	1.000	0.494	0.000	-0.468	0.000

COVID-19 status by lunar phase pairwise comparisons

**Figure 10:**
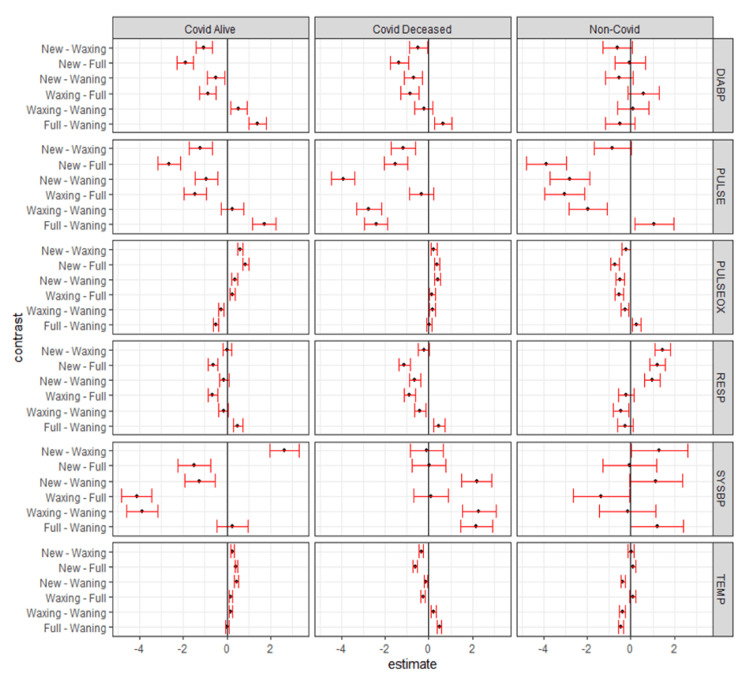
Lunar phase pairwise effect plots and 95% simultaneous confidence intervals by COVID-19 status and vital sign COVID-19: coronavirus disease 2019, DIABP: diastolic blood pressure, PULSE: heart rate, PULSEOX: pulse oximetry, RESP: respiratory rate, SYSBP: systolic blood pressure, TEMP: temperature

The plots in Figure 10 display the pairwise contrast estimates and simultaneous confidence intervals (red) for pairwise differences (contrasts) between COVID-19 statuses by lunar phases and vital signs [18]. If an interval does not contain 0 (vertical line in plots), the contrast is statistically significant. The associated numerical estimates and multiple comparison-adjusted p-values are given in Table 6 [16-18]. As can be seen in Table 6, 22.2% of the p-values of COVID-19 statuses pairwise show a statistical significance of less than 0.05. Unlike lunar phases, there are very few significant differences between COVID-19 statuses. While this may seem incongruous given that their magnitude is larger than those between lunar phases, it is explained by the much larger standard errors for differences between COVID-19 statuses than between lunar phases.

COVID-19 status pairwise comparison by lunar phase for vital signs is shown in Table 6.

**Table 6:** COVID-19 status pairwise comparison by lunar phase for vital signs p-values are adjusted for the 6×3×4=72 (6: vitals, 3: COVID-19 statuses, 4: moon phases) comparisons using the multivariate T-distribution method. COVID-19: coronavirus disease 2019, DIABP: diastolic blood pressure, PULSE: heart rate, PULSEOX: pulse oximetry, RESP: respiratory rate, SYSBP: systolic blood pressure, TEMP: temperature

		New		Waxing		Full		Waning	
Vital sign	Contrast	Est.	p	Est.	p	Est.	p	Est.	p
DIABP	COVID-19 alive - (non-COVID-19)	-1.580	1.000	-1.155	1.000	0.288	1.000	-1.600	1.000
	COVID-19 alive - COVID-19 deceased	2.728	0.976	3.279	0.900	3.276	0.901	2.516	0.988
	COVID-19 deceased - (non-COVID-19)	-4.308	0.553	-4.435	0.512	-2.988	0.945	-4.116	0.623
PULSE	COVID-19 alive - (non-COVID-19)	3.108	0.998	3.474	0.995	1.893	1.000	1.247	1.000
	COVID-19 alive - COVID-19 deceased	2.628	0.999	2.651	0.999	3.778	0.967	-0.360	1.000
	COVID-19 deceased - (non-COVID-19)	0.481	1.000	0.823	1.000	-1.885	1.000	1.607	1.000
PULSEOX	COVID-19 alive - (non-COVID-19)	-0.856	0.706	-1.685	0.006	-2.459	0.000	-1.697	0.006
	COVID-19 alive - COVID-19 deceased	1.459	0.011	1.075	0.183	0.981	0.305	1.502	0.007
	COVID-19 deceased - (non-COVID-19)	-2.314	0.000	-2.760	0.000	-3.441	0.000	-3.199	0.000
RESP	COVID-19 alive - (non-COVID-19)	1.046	0.965	2.478	0.040	2.908	0.007	2.167	0.126
	COVID-19 alive - COVID-19 deceased	-0.533	1.000	-0.788	0.996	-1.014	0.960	-1.037	0.949
	COVID-19 deceased - (non-COVID-19)	1.580	0.635	3.266	0.002	3.922	0.000	3.205	0.003
SYSBP	COVID-19 alive - (non-COVID-19)	-2.443	1.000	-3.782	0.981	-1.012	1.000	-0.054	1.000
	COVID-19 alive - COVID-19 deceased	1.753	1.000	-0.987	1.000	3.258	0.981	5.190	0.611
	COVID-19 deceased - (non-COVID-19)	-4.196	0.941	-2.796	0.999	-4.271	0.935	-5.244	0.759
TEMP	COVID-19 alive - (non-COVID-19)	0.495	0.179	0.253	0.962	0.165	0.999	-0.306	0.856
	COVID-19 alive - COVID-19 deceased	0.950	0.000	0.339	0.660	-0.117	1.000	0.375	0.488
	COVID-19 deceased - (non-COVID-19)	-0.455	0.249	-0.085	1.000	0.281	0.901	-0.680	0.006

 Lunar phase pairwise effect plots and 95% simultaneous confidence intervals by COVID-19 status and vital sign is shown in Figure 10.

As seen in Figure 10, for CD, five out of six pairs are to the left of 0 for DIABP as compared to three each for CS and NC. For CD, all six pairs are to the left of 0 for PULSE as compared to three for CS and five for NC. Within the subset of patients comparing CA to CD from new moon to full moon, DIABP is more negative in CA, PULSEOX is more positive in CA, RESP is more negative in CD, SYSBP is negative in CA compared to others, and TEMP is positive in CA and negative in CD. It can be inferred that the lunar phase pairwise for CD is behaving differently as compared to CS and NC categories. Again, indicating lunar phase variability is seen affecting subsets variably.

In this study, we have seen that the lunar phase does have statistically significant effects on patient vital signs with the most pronounced effects occurring for SYSBP, DIABP, and PULSE. For example, a change of 4 units in systolic blood pressure between the waxing and full moon was statistically significant. Consequently, few point fluctuations caused by the lunar phases will clinically elicit more influence and lead to different outcomes and thus clinical significance as documented by our results in Figure 5-7.

## Discussion

The main finding of this study is that we identified that there were many significant differences in mean vital signs between lunar phases. However, we did not find evidence that specific lunar phases lead to the observed variation, but rather, variation of lunar phases is showing differences in vital signs.

From the data distribution shown in Figure 2, 2%-7% of the total vital signs observations are in the normal range for all three sets of CD, CS, and NC hospitalized patients. Thus, 93%-98% of the vital signs observations are outside the normal window range. Because a majority of these patients' vital signs are already abnormal, a small fluctuation in the abnormal range causes a significant impact. For instance, because 97% of the patient's vital sign observations are outside the normal range, a 3-point difference in systolic blood pressure with respect to the lunar phase is statistically and clinically significant. Theoretically, if this were flipped and 98% of a vital sign observation is in the normal range while 2% of the observations are in the abnormal range, then there has to be a large fluctuation in the 2% of abnormal values to show any statistical or clinical significance.

This vital sign deviation in reference to lunar phases is especially significant in CD patients, followed by a weaker correlation in the CA and NC patients (Figure 3-7). The destabilization windows derived from this variability in vital signs with respect to the lunar phases in CD, CA, and NC patients are able to predict which COVID-19 hospitalized patients can recover.

In Figure 7, for COVID-19 status pairwise, unlike the lunar phase pairwise, there are less significant differences between COVID-19 statuses with reference to moon phases (22% of values less than 0.05). In broad terms, the pattern of effects is similar for COVID-19 alive and COVID-19 deceased patients. It can be inferred that as CS and CD patients are already infected with COVID-19, vital sign variation with respect to lunar phases was not entirely significant. However, PULSEOX and RESP being significant for the NC and CD cohort indicates that COVID-19 viral transmission is happening predominantly through the respiratory medium. Thus, it is affecting those pulmonary vitals, and the variation in those vitals is amplified during the lunar phases compared to non-COVID-19 patients.

Overall, the study results could be explained by a relationship between the different moon phases in relation to circadian rhythms and COVID-19 behavior. Studies show the existence of circalunar rhythms (which refers to the influence of the lunar cycle on internal rhythmic behaviors) that control the reproduction and physiological behaviors of plants, several marine animals such as crabs and certain terrestrial mammals, and humans [19-21]. For example, a study by Dewan et al. found an association between lunar cycles and menstrual cycles [22], while other studies found a link between lunar cycles and birth rates [23,24]. Furthermore, circadian rhythmicity was shown to be controlled by transcription factors such as circadian locomotor output cycles kaput (CLOCK) and brain and muscle Arnt-like protein-1 (BMAL1), which clearly play a role in fertility [25]. Thus, there is growing evidence that circadian and circalunar rhythmicity and reproduction are interconnected.

**Figure 11:**
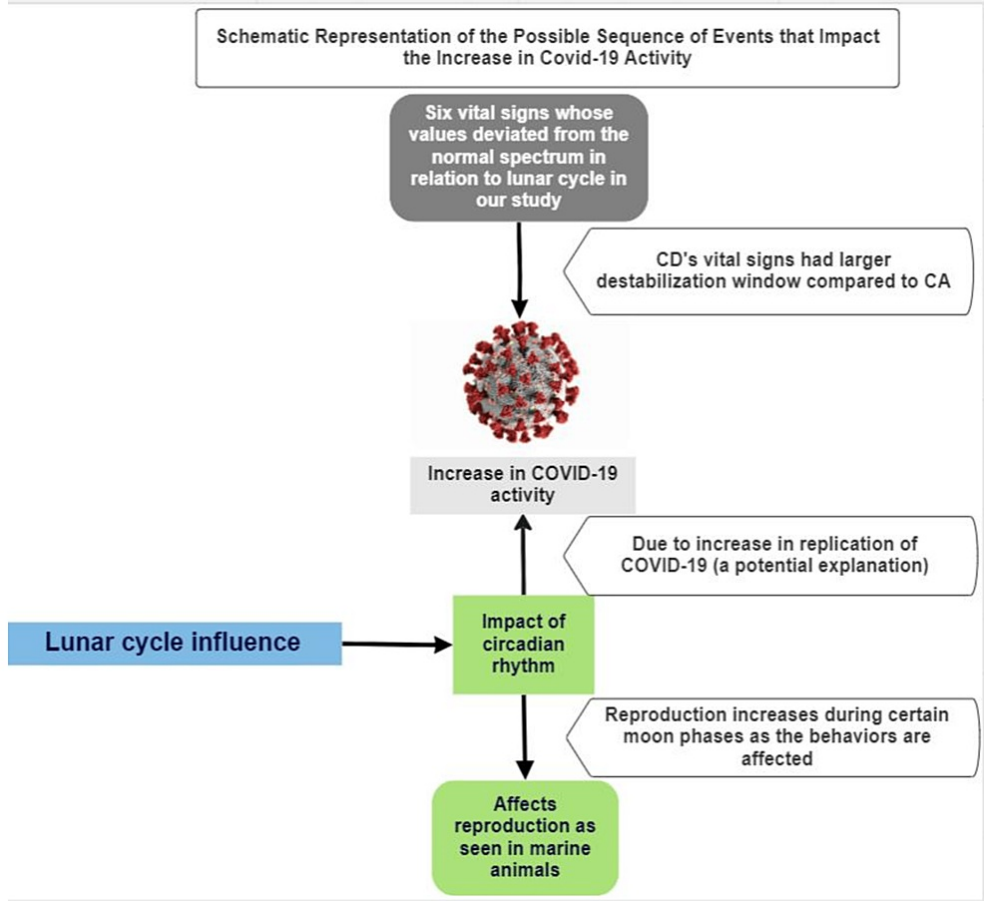
Schematic representation of the possible sequence of events that impact increase COVID-19 activity COVID-19: coronavirus disease 2019, CA: patients who survived from COVID-19 or COVID-19, CD: patients deceased with COVID-19 Image credit: Supriya Koya

There are additional studies that show the relationship between the entry and replication of RNA viruses in humans utilizing the transcription factors of the host that drive circadian gene expression [26]. A study on human lung cells showed that when the transcription factor BMAL1, which is involved in circadian rhythm gene expression, is silenced, COVID-19 virus entry is restricted. This suggests that the COVID-19 virus is using the host’s circadian rhythm-controlling genes that regulate the expression of a viral receptor (ACE receptor) as one of the entry points into the lung epithelial cells [26]. Once the COVID-19 virus enters the cell, it replicates by using the host’s transcription factors. Since these factors control circadian rhythms, it is suggested that COVID-19 is replicating using lunar circadian rhythms. This provides an explanation of why our results showed that COVID-19 patients (CD and CA) compared to non-COVID-19 patients have larger fluctuations in vitals in relation to lunar cycles. COVID-19 is evading the immune system by coupling with essential transcriptional translational feedback loops (TTFLs) [26]. Because TTFLs are involved in homeostasis and immunity [26-28], COVID-19-infected patients also have a harder time normalizing their vital signs and returning to homeostasis. This explanation is depicted in the schematic diagram in Figure 11.

As previously stated, human physiology as reflected by changes in blood pressure (BP), heart rate (HR), and temperature is also influenced by circadian clocks [27]. Therefore, all three sets of CD, CA, and NC patients showed some amount of variability in their vitals in correlation with the lunar phases in the study. It is observed in this study that the difference between maximum and minimum variations of vitals for NC is less than that of CD and CS (Figure 3-7). The range of variation in vital signs likely decreases in NC patients (Figure 3) because more than one vital sign is able to compensate for the imbalances. An example of this process occurs when we are exposed to too much heat and the body’s temperature rises beyond the normal limits. This triggers the autonomic nervous system, which causes sweating and vasodilation to bring the temperature down, thus returning the body to homeostasis. NC patients are likely able to compensate for the variations in vital parameters by the circadian/homeostatic transcriptional and translational feedback loops (TTFLs), which allows for recovery. This recovery was not seen in CD as the gap between the minimum and maximum percentage variation in vital signs was too large to compensate. Hence, CD patients’ vitals were driven into instability. Additionally, in CD patients, TTFLs are disrupted to a greater extent by the COVID-19 virus. It is possible that in CS patients, certain additional host factors are active in recovery and TTFL disruption is not severe, which allows for their recovery. Future studies can focus on and further explain these differences in host factors.

Limitations

This is a retrospective study. The number of vaccinated individuals was not sufficient to draw conclusions with respect to vaccination status. Patients being on ventilators and ECMO could have affected the vital signs, especially measurements of pulse oximetry and respiratory rate. However, using Markov chain Monte Carlo (MCMC) multivariate analysis helped account for multiple confounding factors. Using a larger number of patient datasets can help tease out these variations in pairwise comparisons better. However, this is a pilot study for ascertaining a hypothesis. This study can form the basis for further studies utilizing thousands of patient datasets.

This study overcomes the limitations involved in ascertaining lunar influence on vital signs as seen in prior studies [29]. We are able to compare changes in vitals within the same subjects and inter-patient variability with the help of multivariate mixed model analysis. Having a huge set of data with greater than 200,000 vital data points, this study is able to overcome limitations encountered in other studies. As is the case with other studies that focused on lunar influence, it is hard to exactly measure the biological synchrony due to the influence of human technological interventions and environmental changes [30], but despite the limitations involved, this study is able to account for multiple other variables to show the conclusions as presented here.

## Conclusions

Lunar phases have a significant effect on vital signs in hospitalized COVID-19-deceased, COVID-19-infected, and non-COVID-19 patients. This conclusion is substantiated by the observation that 75.9% of the p-values of the lunar phase pairwise show a statistical significance of less than 0.05, whereas 22.2% of the p-values of COVID-19 status pairwise show a statistical significance of less than 0.05. Patients hospitalized with COVID-19 and deceased from COVID-19 have higher rates of vital sign fluctuations during lunar phases than patients hospitalized without COVID-19.

Range of vital sign fluctuations of DIABP and RR with respect to lunar phases that can help quantitatively estimate the lunar influence on vital signs is derived. This parameter is named the vital parameter destabilization window (DSW). DSW shows using the maximum and minimum varying vital parameters and helps distinguish lunar influence on COVID-19-deceased, COVID-19 alive, and non-COVID-19 patients. DSW shows that when the maximum and minimum vital parameter percentage variation with respect to the normal value as a function of moon phases is between -8.36% and +36.7%, COVID-19 patients are more likely to recover from COVID-19. If the non-COVID-19 destabilization window is taken as a normal window, when the deviation from this window is as much as 36% in COVID-19 patients, recovery is possible. However, if the vital parameters deviate from this window by as much as 60%, recovery is not possible, and patients succumb to COVID-19.

This study helps in the understanding of pathophysiology (as represented by vital parameters) with respect to chronobiology (circalunar rhythms as represented by lunar phases). Monitoring the variability of vital signs with respect to lunar cycles can help identify which patients are more likely to succumb or recover from COVID-19, thus helping maximize the medical care of COVID-19 patients. This pilot study on vital data of 215,220 values serves as a basis to identify treatments to strengthen the circadian-homeostatic feedback loops of COVID-19-infected patients to achieve homeostasis and thus recovery.

## Figures and Tables

**Figure 12:**
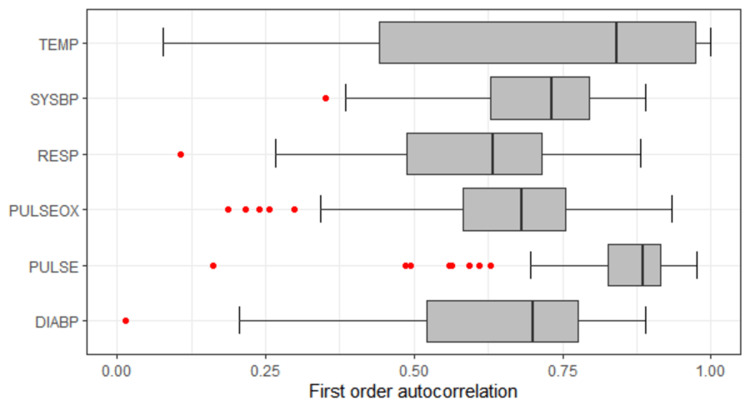
Boxplots of first-order correlations for all vital signs across all patients The thick black line in each box denotes the median and red points outliers. TEMP: temperature, SYSBP: systolic blood pressure, RESP: respiratory rate, PULSEOX: pulse oximetry, PULSE: heart rate, DIABP: diastolic blood pressure

## Appendices

Supplemental figure and table

Figure 12 shows the boxplots of first-order correlations for all vital signs.

The matrix that shows the correlation of vital sign measures within each patient is presented in Table 7.

**Table 7:** Vital sign correlation matrix Bold values are significantly different from 0. DIABP: diastolic blood pressure, PULSE: heart rate, PULSEOX: pulse oximetry, RESP: respiratory rate, SYSBP: systolic blood pressure, TEMP: temperature

	DIABP	PULSE	PULSEOX	RESP	SYSBP	TEMP
DIABP	1.000					
PULSE	0.163	1.000				
PULSEOX	0.001	-0.153	1.000			
RESP	0.132	0.229	-0.115	1.000		
SYSBP	0.483	0.027	-0.096	0.143	1.000	
TEMP	0.012	0.274	-0.027	0.150	0.061	1.000
